# Audit and Re-Audit of Pre-Rapid Tranquillisation (Pre-RT) Workup and Documentation in an Inpatient Psychiatry Ward at Allied Hospital II, Faisalabad.

**DOI:** 10.1192/bjo.2026.11571

**Published:** 2026-06-30

**Authors:** Sammar Fatima, Imtiaz Ahmad Dogar, Duaa Fatima, Palvisha Sajid, Habib Ur Rahman Yazdani

**Affiliations:** Faisalabad Medical University, Faisalabad, Pakistan

## Abstract

**Aims:**

Rapid tranquillisation (RT) is a high-risk intervention used in acute psychiatric settings when de-escalation strategies fail and there is an immediate risk of harm. Best-practice guidelines emphasize comprehensive pre-RT clinical assessment, documentation, and monitoring; however, adherence is often inconsistent, particularly in resource-constrained settings. This audit aimed to assess compliance with established standards for pre-RT workup and documentation and to evaluate the impact of targeted quality improvement interventions through re-audit.

**Methods:**

This was a retrospective, episode-based audit. Forty-two RT episodes over a six-month period (January–June 2025), involving oral or parenteral psychotropic medication administered with the explicit aim of RT, were reviewed against predefined standards. These standards were derived from National Institute for Health and Care Excellence (NICE) guidance, the Maudsley Prescribing Guidelines, and British Association for Psychopharmacology/National Association of Psychiatric Intensive Care Units (BAP/NAPICU) consensus statements. Data were extracted using a structured audit tool. Following baseline analysis, quality improvement measures were implemented, including the introduction of a structured pre-RT checklist and staff education. A re-audit of 28 RT episodes was conducted six months later (July–December 2025).

**Results:**

Baseline compliance with pre-RT standards was variable. Documentation of de-escalation prior to RT was present in 71% of episodes, a clear clinical indication in 83%, and pre-RT clinical workup in 47%, all below target thresholds. Baseline physical observations were documented in 85% of episodes, medication details in 100%, and capacity or consent in 90%, meeting standards. Cardiac/QT risk assessment was documented in 85% of episodes involving haloperidol. However, reassessment prior to repeat dosing was documented in only 42% of episodes, airway and respiratory risk assessment in 78% of benzodiazepine-related episodes, and documentation of contraindications or drug interactions in 66%.

Following intervention, compliance improved across all domains. Documentation of de-escalation increased to 92%, clear indication to 96%, pre-RT clinical workup to 85%, reassessment prior to repeat dosing to 92%, airway and respiratory risk assessment to 92%, and documentation of contraindications to 85%. Medication safety checks, cardiac risk assessments, post-RT monitoring, senior review, and post-incident management planning met or exceeded predefined standards.

**Conclusion:**

This audit identified suboptimal compliance with pre-RT documentation standards, particularly in relation to clinical workup and reassessment. Implementation of a structured checklist and staff education resulted in marked improvements across all measured domains. These findings suggest that focused, low-cost quality improvement interventions can significantly enhance patient safety and documentation standards in resource-limited inpatient psychiatric settings. Ongoing monitoring and periodic re-audit are recommended to sustain improvements.

